# Development and Launch of a Dutch Mobile App (MediMama) on Over-the-Counter Medication Safety During Pregnancy and Breastfeeding: Development and Usability Study

**DOI:** 10.2196/85948

**Published:** 2026-04-02

**Authors:** Veronique YF Maas, Maud de Feijter, Anneke LM Passier, Miranda HM van Tuyl, Agnes C Kant, Maartje Conijn

**Affiliations:** 1 Department Teratalogy Information Service, Netherlands Pharmacovigilance Centre Lareb, Goudsbloemvallei 7, ‘s-Hertogenbosch, 5237 MH, The Netherlands; 2 Department of Clinical Pharmacology and Toxicology, Leiden University Medical Centre, Leiden, The Netherlands

**Keywords:** medicines, pregnancy, breastfeeding, lactation, mobile app, development, safety, information, over-the-counter medicines.

## Abstract

**Background:**

Over-the-counter (OTC) medicines are frequently used during pregnancy. As these medicines are often used without medical supervision, accessible and reliable safety information is essential. However, finding reliable and understandable information on the safety of these medicines during pregnancy is often experienced as difficult. Hence, there is a need for a new easily accessible electronic health (eHealth) tool that empowers women to actively seek information to support safer self-medication practices during pregnancy and breastfeeding.

**Objective:**

This study aimed to describe the development and dissemination process of a Dutch mobile app providing reliable safety information on OTC medicines during pregnancy and breastfeeding using a development and formative evaluation approach.

**Methods:**

The app was developed over a 2-year project comprising 5 phases, including preparation, development, preimplementation, implementation, and evaluation. Mixed-method strategies, including questionnaires, focus groups, and user feedback rounds, were applied to involve the target population in the development process. Medicine safety information in the app was based on the latest scientific evidence. First-year app-usage outcomes included app downloads, usage patterns, and information-seeking behavior.

**Results:**

Input from 253 potential users formed the foundation for the development of the MediMama app (Netherlands Pharmacovigilance Centre Lareb), with users expressing a need for clear, reliable, and easily accessible information on medication safety during pregnancy and breastfeeding. The app was launched on Mother’s Day 2024 and provides safety information on over 250 OTC medicines, including supplements and herbal remedies, across 27 medicine categories. Promotion occurred through multiple online and offline channels. During its first year, the MediMama app was downloaded 22,415 times, with an average of 370 unique daily users, indicating substantial user engagement. Information on paracetamol (acetaminophen) and nasal sprays was most frequently accessed, reflecting the need for information on commonly used OTC medicines among the target population.

**Conclusions:**

One year after its launch, the MediMama app is considered a promising tool in maternity care, meeting the target population’s need for accessible OTC medicine safety information. The app aims to support informed decision-making, contributing to safer medication use during pregnancy and breastfeeding. Further research is required to evaluate the effectiveness of the implementation strategy, as well as the app’s impact on maternal medication use behaviors and health outcomes.

## Introduction

Medication use during pregnancy and breastfeeding is common, with an estimated 80%‐90% of women reporting the use of at least one (prescribed) medicine during pregnancy [1,2]. Nonprescription or over-the-counter (OTC) medicines are frequently used to manage pregnancy-related or seasonal symptoms such as heartburn, pain, or cold symptoms [3,4]. Although readily available in supermarkets and drugstores, OTC medicines are not without risk. Inappropriate use of some OTC medicines may increase the risk of congenital anomalies, adverse perinatal outcomes, and long-term health consequences for the offspring [3,5]. For instance, use of nonsteroidal anti-inflammatory drugs such as ibuprofen or naproxen in the second half of pregnancy has been associated with higher risks of constriction of the ductus arteriosus, oligohydramnios, and fetal renal dysfunction [6-8]. Similarly, the use of certain antihistamines during breastfeeding may reduce milk supply or cause sedation and irritability in infants, especially at high doses or with prolonged use [9,10]. While some women may be unaware of these potential risks, others tend to overestimate teratogenic risks and may be reluctant to use necessary medications to manage health conditions (eg, fever or pain during pregnancy), which in some cases could result in greater harm than the medications themselves [11,12].

Empowering women to make informed medication choices is therefore essential. Compared with prescription medicines, OTC medicines are widely available, less stringently regulated, and frequently used without professional guidance [3]. This shifts the responsibility for safety assessment to the individual user. Uninformed decisions about taking OTC medicines (including herbs, homeopathic remedies, and supplements) may lead to the usage of unsafe medicines without the user even being aware. While teratogenic risk information is available online, it has been suggested to be unclear, hard to find, or challenging to interpret for the lay audience. Hence, new interventions tailored to the target population are needed. Electronic health (eHealth) apps are described as the next generation in prenatal care and have been demonstrated to improve antenatal care quality, medication adherence, and pregnancy outcomes [13-16]. Research suggests that up to 95% of pregnant women search online for health information [17,18]. Mobile apps have become one of the primary sources of information and tend to rapidly replace traditional methods of obtaining information from health professionals and written resources [13,19-21]. Consequently, there are more apps available with pregnancy-related information compared to any other medical topic at the time of this writing [22].

While online information is easily accessible for women of reproductive age, it also entails risks, particularly regarding the reliability and accuracy of online sources [23,24]. Conflicting information from multiple online sources is common and can result in women seeking additional unreliable sources, experiencing increased anxiety or even avoiding necessary medication [18,25,26]. For example, previous research suggests that the main reasons women are hesitant to take medication during lactation include fear of harming the infant, lack of clear and consistent information, and conflicting advice (eg, between health care providers and the often outdated and overly restrictive information provided in the product label) [27-31]. This can lead women to discontinue breastfeeding or avoid necessary medication, even though most medications are compatible with breastfeeding [32,33]. Therefore, it is important to develop a single tool that is integrated across all stages of maternity care, providing reliable, clear, and comprehensible safety information.

Although several apps providing medication safety information for pregnancy or breastfeeding have been developed, awareness and use of these tools remain suboptimal, as they are often not easily accessible or widely known to users [34-36]. In addition, since brands of OTC medicines, use patterns, and regulatory recommendations on safety during pregnancy and lactation differ between countries, each country bears responsibility for providing, maintaining, and effectively disseminating locally relevant recommendations on medication safety [37,38]. In response to these challenges, this study aims to describe the development and dissemination process of a free and easily accessible Dutch mobile app on the safety of OTC medicines during pregnancy and breastfeeding.

## Methods

### Overview

**Figure 1.**
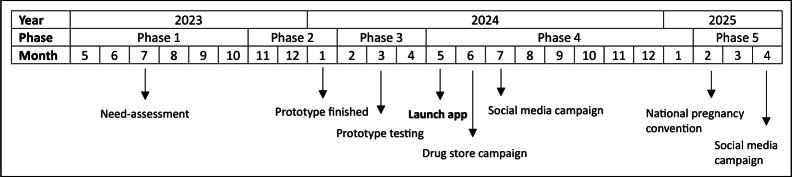
Overview of the phases and activities within the app development process.

The app is developed by the Dutch Teratology Information Service (TIS), part of Pharmacovigilance Centre Lareb. The Dutch TIS is the national knowledge center for medication safety during pregnancy and breastfeeding [39]. TIS provides evidence-based information on over 1100 (primarily prescription) drugs through a knowledge bank on its public website, which receives approximately 1.5 million visits annually [40]. Next to the knowledge bank, there was a desire for easier and faster access to information on OTC medications, for example, when standing in the drugstore. Inspired by a widely used Dutch app on the safety of food during pregnancy (the ZwangerHap app; The Netherlands Nutrition Centre) [41], a grant proposal for the development of a new app on the safety of OTC medication was submitted to and approved by the Netherlands Organization for Health Research and Development (ZonMw; grant number: 10140162210007). The 2-year development and implementation project started on May 1, 2023. This study followed a development and formative evaluation approach in which data were collected through questionnaires and focus groups to iteratively refine the new mobile app. This study followed 5 consecutive phases, including preparation, development, preimplementation, implementation, and evaluation (Figure 1). The month numbers displayed in Figure 1 represent the month of the year.

### Phase 1. Preparation Phase

First, a project group consisting of representatives from the professional associations of midwives and gynecologists, as well as a delegate from the umbrella organizations representing drugstores, was assembled. Next, a needs assessment was conducted among the target population using 2 tailored online questionnaires, one for women who were pregnant or gave birth within the past year and one for health care professionals working with this group. These were the only inclusion criteria for participating in the needs assessment. Questions focused on information-seeking behavior, content preferences, and distribution strategies. Given their brief and exploratory nature, the questionnaires primarily assessed opinions and prior behaviors and were therefore not pilot-tested nor formally validated. A translated version of one of the distributed questionnaires is provided as supplemental material. Participants were recruited using convenience and snowball sampling approaches, primarily through the Dutch Pregnancy Drug Registry (as part of the Dutch TIS), social media, an umbrella organization of drugstores, and the TIS’s professional network. Together, these recruitment strategies aimed to provide a comprehensive representation of the target population, thereby reducing the risk of sampling bias. The target sample size of 100 completed questionnaires was determined based on feasibility considerations and the exploratory aims of the study. The completed questionnaires were analyzed descriptively and extensively discussed within the project group, and the outcomes were used as a basis for the development phase.

### Phase 2. Development Phase

During the development phase, an initial draft of the app was devised together with a marketing agency and information technology partner. When developing the app prototype, insights from the needs assessment were used, and discussions with the project group addressed which barriers health care professionals needed to overcome before recommending the app. Key decisions included the navigation structure, type of information, required warnings, interface design, app name, and integration with the existing TIS knowledge bank on the website for extended content. A comprehensive list of OTC medicines, such as analgesics, antacids, vitamins, and herbal supplements, was based on the most commonly used OTC medicines during pregnancy in the Dutch Pregnancy Drug Registry and further extended with OTC medicines identified during field visits to pharmacies, drugstores, and supermarkets. Medicine safety information included in the app was drafted by teratology experts from the Dutch TIS, based on the latest scientific available evidence. Content drafting was supervised by a medical doctor and a pharmacist. All displayed content was fully managed in-house, allowing for continuous updates and modifications as needed. Finally, in consultation with legal experts, privacy terms and disclaimers were carefully drafted. By the end of this phase, a fully functional app prototype was completed.

### Phase 3. Preimplementation Phase

Before the launch, a prototype of the app was extensively tested among the target population using a mixed method approach by collecting both qualitative data (focus groups) and quantitative data (questionnaires). First, focus groups were conducted consisting of target users and teratology experts. Participants were recruited through convenience and snowball-based approaches, primarily via midwifery practices, pregnancy fitness classes, and professional networks. Each focus group was scheduled for a maximum of one hour. Participants first completed exercises designed to facilitate navigation through the app, after which the facilitator led a group discussion following a predesigned script, eliciting feedback aimed at improving the app. Sessions were audio-recorded with consent, and all feedback points were documented and descriptively analyzed without the use of an analytical framework. We aimed to conduct 5 focus groups with a minimum of 5 participants each, a target determined pragmatically in light of feasibility and the short data collection timeframe. Subsequently, an online questionnaire, mirroring the focus group tasks, was distributed among health care providers and women who were pregnant or gave birth within the past year. These were the only inclusion criteria for administering the questionnaire. The aim was to collect 10 completed questionnaire responses. All feedback from the focus groups and questionnaires was evaluated, and final adjustments to the app were made accordingly.

### Phase 4. Implementation Phase

The launch of the app was supported by both broad and targeted communication campaigns. Dissemination relied on an informing strategy, engaging stakeholders to promote the app through online and offline channels. Health care professionals were positioned as key figures by recommending the app during consultations, as part of a motivational approach. Booster campaigns were periodically initiated to reach the continuously changing target population. All feedback received from app users was carefully reviewed, and whenever possible, immediate additions or changes were made to the content.

### Phase 5. Evaluation Phase

Evaluation metrics included the number of app downloads, App Store ratings, user feedback, number of daily active users, most searched medications, and total number of page views. Tracking of anonymous in-app user behavior commenced 5 months postlaunch, following an app release. A cautious initial target of 15,000 downloads was set for the first year, based on the approximate annual birth rate of 170,000 in the Netherlands, with the understanding that awareness and adoption would take time to build [42].

### Ethical Considerations

This study did not require approval from a medical ethics review committee, as it did not qualify as medical research involving human subjects under the Dutch Medical Research Involving Human Subjects Act. All collected data were anonymized before analysis to protect participant privacy and confidentiality. Participation was voluntary, and informed consent was obtained from all participants prior to participation. Only focus group participants received a gift voucher of their choice as a token of appreciation for their time and contribution.

## Results

### Phase 1. Preparation Phase

A total of 217 individuals completed the needs assessment questionnaire; 184 (84.8%) were women who were pregnant or had given birth within the past year, and 33 (15.2%) were health care professionals (Table 1). Among the women, 160 out of 184 (87.0%) were pregnant at the moment of administering the questionnaire (mainly in the second trimester), and 10 out of the 24 women who recently gave birth (41.7%) were still breastfeeding. The majority of women (134/184, 72.8%) reported actively seeking safety information before using any medication. The need for information on OTC medicines (142/184, 77.2%) and health products (eg, supplements, herbs, and vitamins; 122/184, 66.3%) was higher than the need for information on prescribed medicines (70/184, 38.0%). Preferred sources of information were official (government) websites (151/184, 82.1%), product information (125/184, 67.9%), and health care providers (115/184, 62.5%). The primary motivation for seeking safety information was (1) to determine whether a medicine can be used during pregnancy and breastfeeding or not (2), which medicine is the preferred treatment option for specific (pregnancy) complaints, and (3) what the possible adverse drug reactions are. Open-ended responses underscored challenges in finding trustworthy and accessible information, and frustration with inconsistent recommendations across sources.

**Table 1.** Results of the needs-assessment questionnaires conducted in the preparation phase data are presented as n (%).

Characteristic	Value
Women (n=184)	
Health status, n (%)	
Pregnant, 1st trimester	34 (18.5)
Pregnant, 2nd trimester	89 (48.4)
Pregnant, 3rd trimester	37 (20.1)
Gave birth <1 year, still breastfeeding	10 (5.4)
Gave birth <1 year, not breastfeeding	14 (7.6)
First-time pregnancy, n (%)	
Yes	97 (52.7)
No	87 (47.3)
Frequency of information-seeking behavior before medicine use, n (%)	
Always looks up information	134 (72.8)
Often looks up information	19 (10.3)
Sometimes, only when in doubt	26 (14.1)
(Almost) never	5 (2.7)
Searched information on^a^, n (%)	
OTC medicines^b^	142 (77.2)
Other health products^c^	122 (66.3)
Prescribed medicines	70 (38.0)
No need for more information	30 (16.3)
Preferred channel to receive information^a^, n (%)	
Online (neutral evidence-based medicine websites)	151 (82.1)
Product information (medicine leaflet)	125 (67.9)
Health care provider	115 (62.5)
Online (pregnancy websites or forums)	92 (50.0)
Shop assistant	28 (15.2)
Family and friends	15 (8.2)
Online pregnancy forum	13 (7.1)
Likelihood of using a new mobile app for OTC medicines^d^	
Mean (SD)	7.8 (2.3)
Score≥7	146 (79.3)
Health care professionals (n=33)	
Profession, n (%)	
Drug store employee	20 (60.6)
Pharmacist	5 (15.2)
Midwife	3 (9.1)
Assistant pharmacist	2 (6.1)
Other	3 (9.1)
Will your job be easier if women use a new mobile app for OTC medicines^e^	
Mean (SD)	6.0 (2.1)
Score≥7	16 (48.4)
Likelihood of using a new mobile app for OTC medicines^d^	
Mean (SD)	6.0 (3.5)
Score≥7	20 (60.6)
Likelihood of recommending a new mobile app for OTC medicines^d^	
Mean (SD)	7.3 (2.4)
Score≥7	25 (75.8)

a Multiple answers possible.

b OTC medicines: over-the-counter medicines.

c The exact answer option was “Information about health products other than OTC medicines (eg, supplements, vitamins, and herbal remedies)”.

d On a scale of 1 (very unlikely) to 10 (very likely).

e On a scale of 1 (Totally disagree) to 10 (totally agree).

Health care professionals (n=33) reported using the knowledge bank of TIS on the website frequently and appreciated the clear distinction between pregnancy and breastfeeding guidance. The main reasons for seeking safety information were (1) to determine whether or not a medicine can be used during pregnancy and breastfeeding (2), what the possible adverse drug reactions are, and (3) which medicine is the preferred treatment option for specific (pregnancy) complaints. While some believed the app could reduce their workload, others expected women would still seek personal advice. Finally, health care professionals were moderately likely to use the app themselves (mean 6.0, SD 3.5) but more likely to recommend it to patients (mean 7.3, SD 2.4 out of a scale from 1 to 10).

### Phase 2. Development Phase

**Figure 2.**
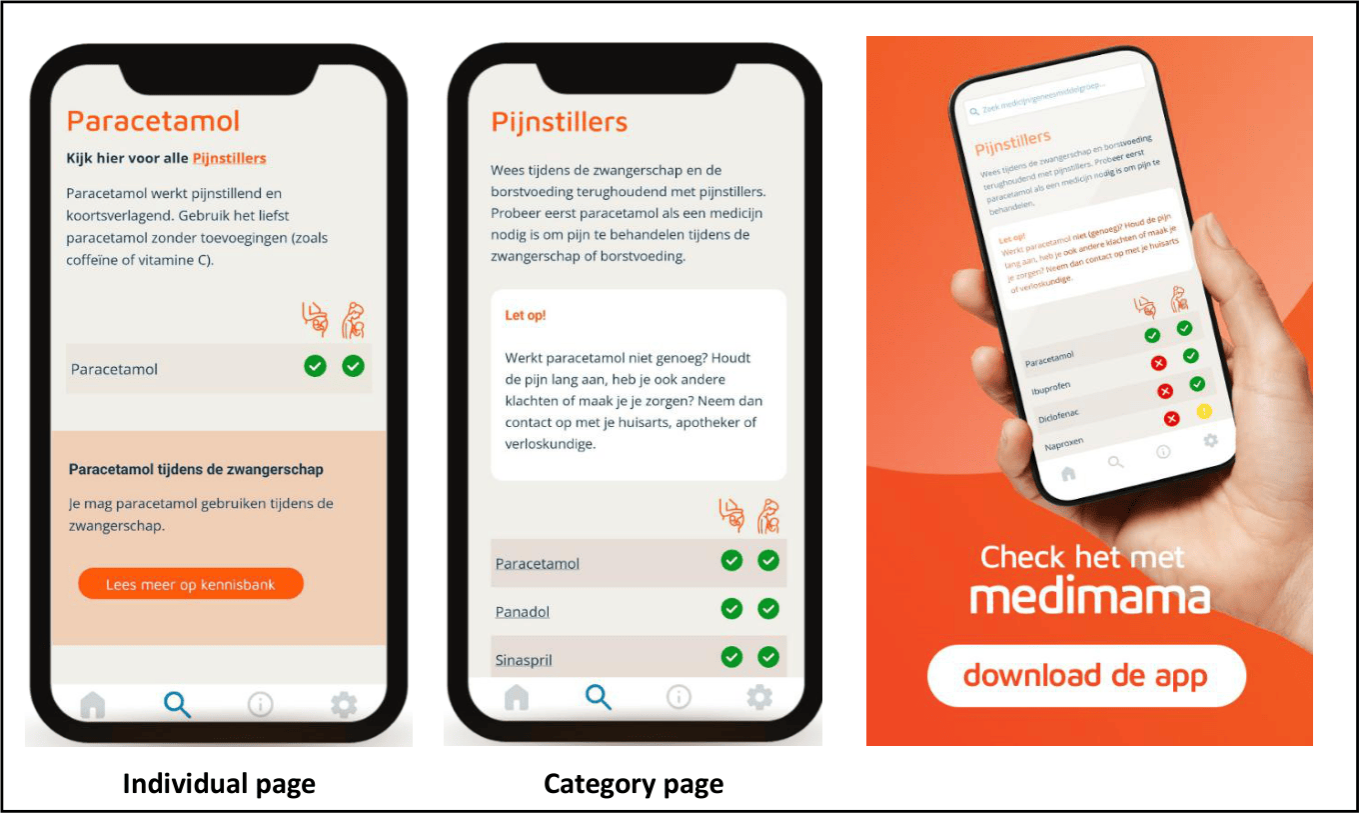
Overview of the different pages and the look and feel of the MediMama app.

The primary focus in the user experience testing was to make sure that the required information was found as quickly as possible and was comprehensive and easy to understand. For this purpose, all medicines in the app are classified within 3 risk categories using the traffic light colors, including green (can be used), yellow (be careful and read more information), and red (not to be used). Safety information in the app is distributed through 2 kinds of information pages (Figure 2):

1. An individual page contains short and easily understandable product-specific safety and usage information. The app contains 260 individual pages, including medicines, products, vitamins, and herbals. On all individual pages, a referral button to the specific information on the TIS website is placed for more detailed information.
2. The category page provides an overview of a group of medicines or products where all risk categories from all relevant medicines are displayed in a table. The app contains 28 category pages, for instance, analgesics, antacids, or hay fever tablets. Additionally, this page provides lifestyle recommendations to relieve the (pregnancy) complaint before the initiation of medication.

Users can search by active ingredient, brand name, or health complaints. The app name, MediMama (Netherlands Pharmacovigilance Centre Lareb), was selected in collaboration with followers of a large Dutch pregnancy platform. The look and feel of the MediMama app are displayed in Figure 2. Based on user preferences, the app was developed as a native app as well as a web-based version.

### Phase 3. Preimplementation Phase

The prototype was tested in 5 focus groups (n=23 participants) and via an online questionnaire (n=14; Table 2). During the focus groups, thematic saturation was observed. Participants in the focus groups reported that the app was easy to use, had an appealing look and feel, and the displayed safety information with the 3 colors was a big plus. Minor technical issues were noted on certain devices. Key suggestions included shortening texts, improving ease of reading, removing account registration, improving the disclaimer text, and considering adding more information about medication usage, such as duration and dosage. The online questionnaire was completed by 6 out of 14 (42.9%) women who were pregnant during the administration of the questionnaire or gave birth within one year of completing the questionnaire and 8 out of 14 (57.1%) health care providers with different backgrounds (Table 2). All questionnaire respondents rated the app’s ease of use ≥8/10. Most women reported that they would probably (3/14, 21.4%) or definitely (11/14, 78.6%) use or recommend the app. Suggestions were made to improve specific texts (eg, the disclaimer) and to add references to other sources of information for more detailed information on, for example, medication usage or the health condition. All health care providers agreed with the statement that the information in the app is appropriate and suitable for women who are pregnant or breastfeeding.

**Table 2.** Results of the mixed methods prototype testing of the MediMama app conducted in the preimplementation phase.

Category	Findings
Focus groups (n=23 participants)	
Focus group sessions	
Online (team session)	Teratology experts (n=9)
Local gym	Pregnant women or women who gave birth <1 year (n=4)
Online	Pregnant women or women who gave birth <1 year (n=3)
Online	Women who gave birth <1 year (n=2)
Midwifery practice	Pregnant women (n=4) and midwife (n=1)
Examples of technical issues	Disclaimer page crashed on some devices; formatting inconsistencies across devices; design differences between dark and light phone settings
Examples of positive aspects	Appealing layout and app name; easy to search for information; positive feedback on referrals to other platforms for more detailed information
Examples of suggestions to improve	Texts could be shorter and include less jargon; remove the option of creating a personal profile; include the category page in search results when searching for an individual medicine; onboarding information can be overwhelming
Online questionnaires (n=14 participants)	
Target population	
Pregnant	Second trimester (n=1); third trimester (n=2)
Gave birth <1 year	Breastfeeding (n=2); not breastfeeding anymore (n=1)
General practitioner	3
Midwife	2
Other HCP^a^	3
MediMama app easy to use^b^	
Mean	8.6
Score ≥8	14 (100)
Examples of positive aspects	Clear, short, and understandable information; simple design with clear symbols and colors; clear distinction between pregnancy and breastfeeding; useful referrals to other platforms for more detailed information
Examples of suggestions to improve	Improve information in the disclaimer and onboarding; explicitly refer users to the prescribing doctor for information on prescribed medication; consider including preconception recommendations; be cautious when categorizing all vitamins as “green” (safe to use)
Would you use or recommend the MediMama app?	
Definitely not or maybe	0 (0)
Probably	3 (21.4)
Definitely	11 (78.6)

a HCP: health care professional.

b On a scale from 1 (difficult to use) to 10 (very easy to use)*.*

### Phase 4. Implementation Phase

The MediMama app was launched on Mother’s Day (May 12th), 2024, and has been freely available in the app stores since then [43,44]. During the first year, the MediMama app was promoted through various channels, with the 4 main promotional strategies being the following:

1. Drug store campaign: in over 800 drug stores, MediMama “shelf cards” were displayed. This generated significant visibility and raised awareness among store employees and visitors.
2. Online social media campaign: targeted campaigns via Instagram (Meta Platforms), Facebook (Meta Platforms), and Google Play (Google Inc) were conducted. Campaigns were updated every few weeks to optimize reach. These platforms use advanced algorithms and machine learning to optimize advertorial performance.
3. Dutch pregnancy convention: over 30,000 (pregnant) visitors attended a 4-day Dutch pregnancy convention. At which the MediMama app was promoted through a booth, daily master classes, and giveaways.
4. To reach health care providers from various disciplines, the development and launch of the app were promoted through channels as presentations at national medical conferences, posters and flyers in waiting rooms, local health care provider meetings, publications in professional journals, and news items from several professional associations.

User feedback led to several app improvements. In response to frequent requests, the most searched prescription medicines were also added. The prescribed medicines in the app all receive the “yellow risk category” (be careful and read more information) and directly refer to the medicine-specific information on the TIS website for more detailed information and the advice to discuss this medication use with their physician.

### Phase 5. Evaluation Phase

**Figure 3.**
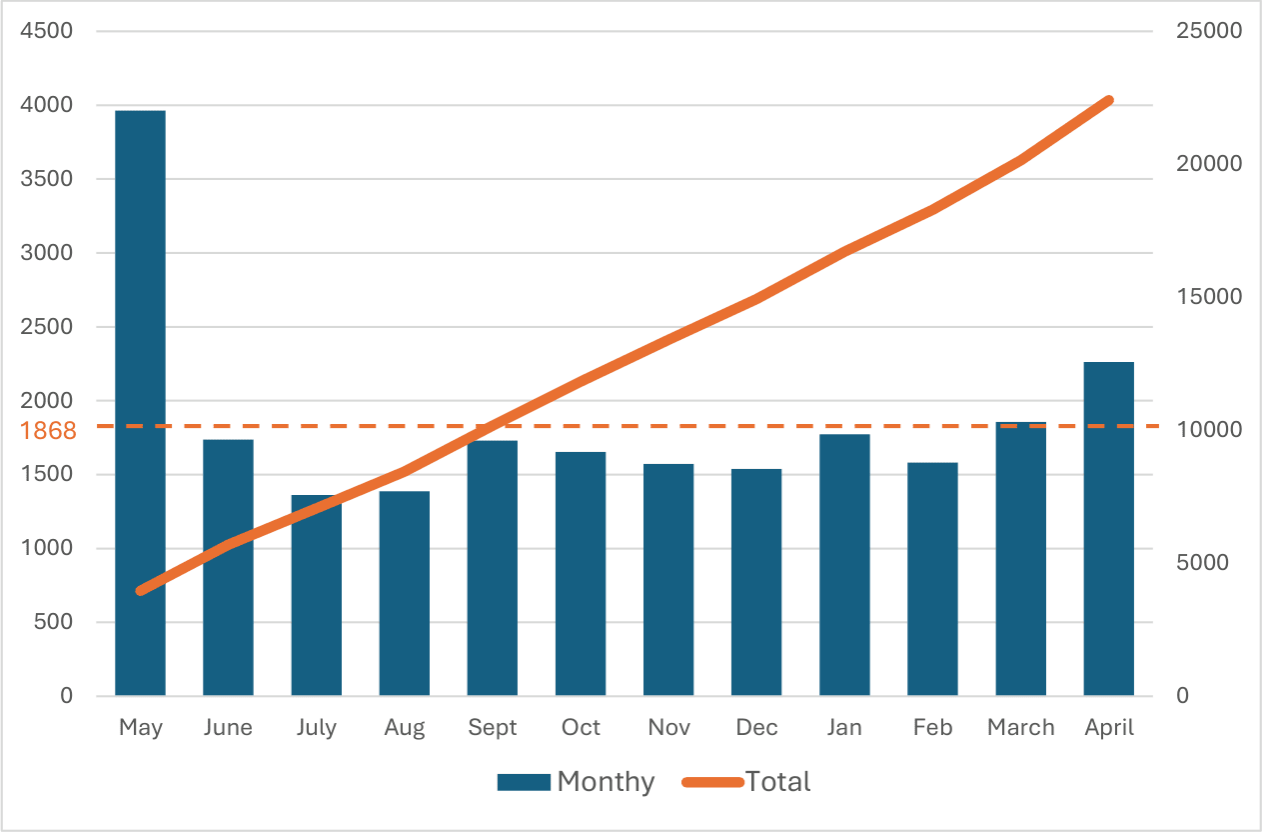
Overview of the MediMama downloads within the first year. The left Y-axis scale represents the number of monthly downloads (blue); the right Y-axis scale represents the total number of downloads (orange); the orange dotted line represents the average number of monthly downloads; the numbers within the blue bars represent the average number of daily downloads.

**Figure 4.**
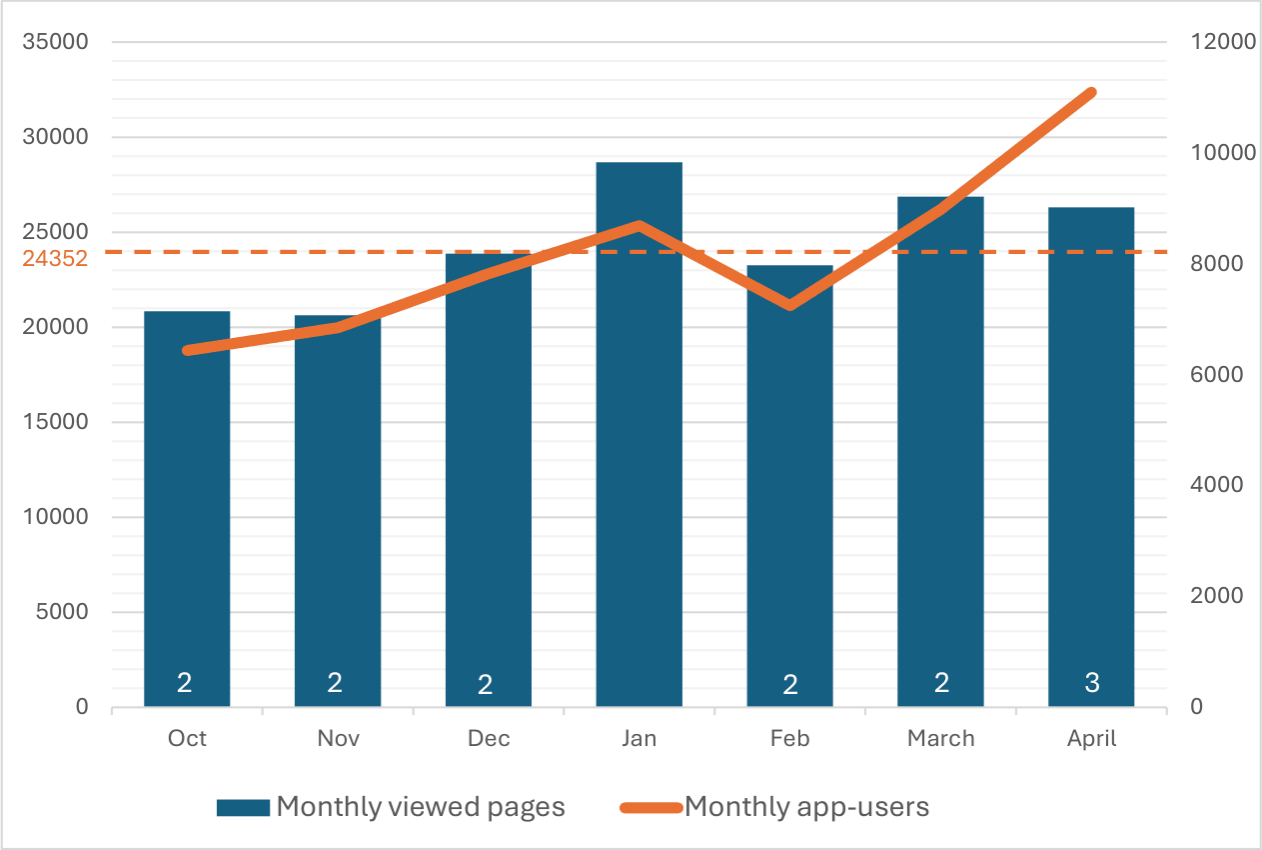
Overview of the MediMama users. The left Y-axis scale represents the number of monthly views of all MediMama pages within the app (blue); the right Y-axis scale represents the monthly number of app users (orange); the dotted orange line represents the average number of monthly page views; the numbers within the blue bars represent the average number of daily app users.

**Figure 5.**
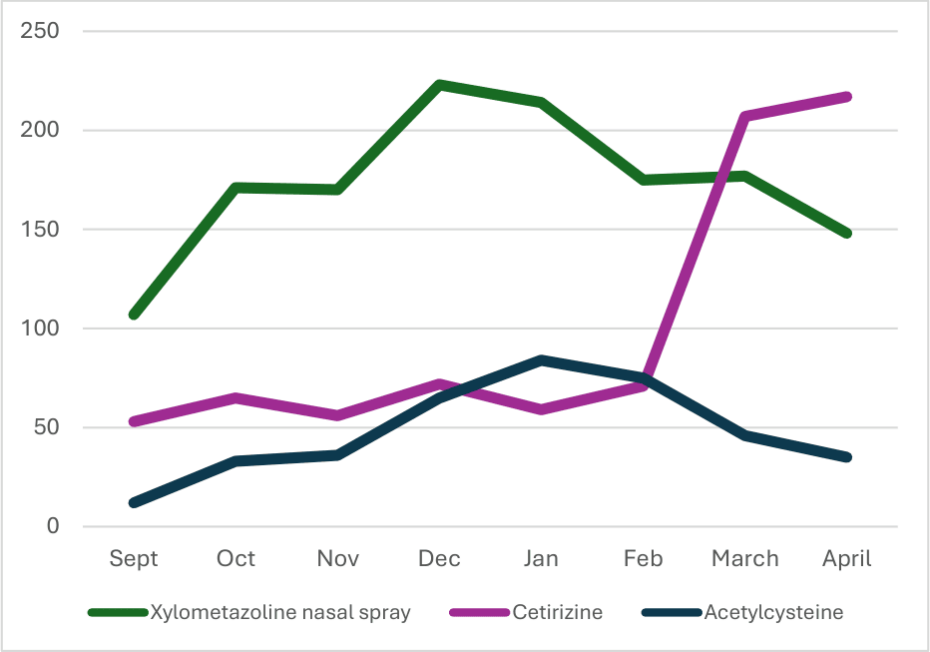
Examples of seasonal trends in search terms for 3 medicines in the MediMama app. Numbers correspond with the monthly number of times xylometazoline nasal spray, cetirizine (hay fever medicine), and acetylcysteine (cough syrup) were searched.

In its first year, the app was downloaded 22,415 times, averaging 1868 downloads per month (Figure 3). Peak activity occurred in the launch month (n=3963). After the initial peak of reaching the early adopters, the average daily number of downloads gradually increased from 44 per day up to 75 per day. The social media campaign proved to be one of the most effective strategies to reach the target population. On average, app users viewed 3 pages per session; in total, on average, 24,352 pages were viewed each month (Figure 4). The number of daily app users increased from 207 per day to 370 daily app users over the course of 6 months. However, the growth in the number of downloads and app users did not follow a consistent upward trend each month over the course of the first year, resulting in some fluctuation in usage (eg, the short month of February or seasonal changes). To date, the MediMama app has received 11 reviews across both app stores, all of which have awarded it the maximum rating of 5 stars. Most of the feedback received via email and social media concerned the need for the app to be available in multiple languages and to include information on prescribed medications. Paracetamol (acetaminophen) and nasal sprays turned out to be the most frequently searched and viewed medicines in the app (Table 3). While category pages received more overall views, individual pages were more frequently accessed via search. This indicates a distinction in user behavior; searches were primarily aimed at specific medicines, whereas the higher number of views on category pages suggests that users actively navigated through the app to gain a more comprehensive understanding of an entire group of medicines. Finally, seasonal trends in search terms are visible corresponding with the seasonal health complaints (Figure 5). For example, in the flu season (December-February), information-seeking behaviors leaned toward medicines like nasal sprays or cough syrups, while a peak in hay fever medicine (eg, cetirizine tablets) was noticeable from March onward.

**Table 3.** Top 10 most searched terms and viewed medicine pages in the MediMama app.

Most viewed		Top	Most searched	
Type^a^	Title		Title	Type
I	Paracetamol^b^	1	Paracetamol^b^	I
C	Nasal sprays for colds	2	Nasal sprays for colds	C
C	Sore throat relievers	3	Ibuprofen	I
I	Xylometazoline nasal spray	4	Cough remedies	C
C	Cough remedies	5	Strepsils cough drop	I
C	Painkillers	6	Antacids	C
C	Antacids	7	Rennie^c^	I
I	Ibuprofen	8	Sore throat relievers	C
I	Strepsils cough drop	9	Xylometazoline nasal spray	I
C	Hemorrhoid remedies	10	Tablets for allergy and hay fever	C

a Either an individual page (I) or a category page (C)

b Also known as acetaminophen.

c Brandname of an antacids containing calcium carbonate and magnesium carbonate.

## Discussion

### Key Findings

This study details the development and dissemination process of MediMama, a Dutch mobile app providing evidence-based information on the safety of OTC medicines and products during pregnancy and breastfeeding. Preimplementation research suggested that the preferred route for receiving safety information on medicine was online and emphasized the need for a new mobile app. Involving the target population throughout development ensured alignment with user needs. One year postlaunch, the MediMama app is considered a promising eHealth tool, addressing the demand for accessible, reliable OTC medicine safety information in maternity care.

### Comparison to the Literature

Previous eHealth apps in maternity care have demonstrated a successful tool in improving safe medication use and overall maternal health outcomes during pregnancy and lactation [15]. Educational apps and clinical decision support tools have been shown to improve medication adherence, increase antenatal care attendance, and support behavioral changes for topics such as healthy diet, smoking cessation, and vaccination uptake [16,45-47]. Usability and acceptability of mobile health apps during pregnancy are generally high, with pregnant women and health care providers reporting satisfaction and willingness to recommend these tools [16,45,48,49]. Some previously developed apps on the safety of medication use during pregnancy or breastfeeding have been developed, for instance, LactSafe (NIH LactMed - MotherToBaby (OTIS)) [34], MommyMeds (InfantRisk Center) [35], and Meds4Mums2B (Innovative Health Initiative (IHI) ConcePTION, in collaboration with the Medicines and Healthcare products Regulatory Agency (MHRA)) [36]. The MediMama app differs from existing tools by being freely accessible worldwide, providing comprehensive safety information for both pregnancy and breastfeeding based on scientific literature. In addition, the MediMama app has been scientifically evaluated, and data on usability and user satisfaction are available. Because of limited available evidence, the effectiveness of mobile health apps in improving maternal knowledge, behavior, and perinatal outcomes remains inconclusive [45,50].

Despite their potential, eHealth tools during pregnancy also carry certain risks with regard to self-treatment [19]. Self-medication during pregnancy is prevalent and can lead to inappropriate drug selection, misdiagnosis, dosing errors, delayed medical help–seeking, and inefficient treatment which may result in adverse maternal and fetal outcomes [51,52]. The MediMama app aims to mitigate these risks by using intuitive, color-coded guidance that is immediately interpretable at a glance and by advising on how to treat a complaint through lifestyle changes instead of medicines (ie, for heartburn). With regard to the quality of eHealth apps, previous reviews elaborated on the growing concern over the quality of several (commercial) pregnancy apps with regard to poor or unclear evidence base of content, lack of expert involvement, and poor validation [19,53-56]. Emerging artificial intelligence–based health tools further complicate this landscape, with risks of misinformation due to limited contextualization and transparency [57,58]. To navigate these waters of conflicting health information, health care providers can play a central role in advocating for evidence-based trustworthy health information provided by noncommercial public institutions.

Pregnancy and breastfeeding represent periods of heightened health awareness, yet implementation of interventions is challenging due to the dynamic and transient target population [59,60]. The relatively short duration of pregnancy and the subsequent breastfeeding period means that the target group is constantly changing, with individuals entering and exiting the eligible target population in a matter of months. During the implementation phase of the MediMama app, we also experienced the considerable effort it required to reach this constantly changing target population. We have experienced that focusing on online marketing and involving health care providers in disseminating the app could be a promising and sustainable approach to reaching this constantly changing target population.

### Strengths and Limitations

This is the first study to systematically describe the development and dissemination process of a mobile app focused on medicine safety in pregnancy and breastfeeding, using mixed methods to capture user and provider perspectives. The diverse set of offline and online strategies used to distribute the MediMama app enabled us to reach this constantly changing target population. Due to this multifaceted promotional approach, the target aim of 15,000 downloads within the first year was reached within the first 7 months. The MediMama app has been well received; the number of daily users keeps increasing, and reviewers praise its simplicity, trustworthiness of the information, and their privacy protection since the app is used anonymously.

However, anonymity limits evaluation of user demographics, hindering assessment of whether the app reaches intended subgroups (eg, pregnant women vs health care providers) and its impact on clinical workload. Consequently, it is difficult to assess whether the app conveys the appropriate tone of voice in presenting safety information. Further user research is therefore needed. Another limitation of our study is that the preimplementation recruitment via the pregnancy registry may have introduced selection bias and the use of unvalidated and not pilot-tested questionnaires to assess user needs. This may have affected the quality and reliability of the collected data. However, the questionnaires were developed in close alignment with the study objectives, supporting their face validity despite the lack of formal validation. Finally, most suggested feedback after the launch of MediMama was making the information in the app available in multiple languages. By limiting the language options to Dutch only, we were likely unable to reach some of the most vulnerable populations, including individuals with limited digital literacy or access, who might benefit most from health guidance. However, vulnerable pregnant women may require additional and fundamentally different forms of health education because a “one-size-fits-all” approach is unlikely to reach a heterogeneous pregnant population with varying levels of health literacy [60].

### Future Research

Prior to the large-scale implementation of mobile health apps during pregnancy, more research is needed on their effectiveness. As previous studies suggested, there is still much to learn on the impact of mobile health apps during pregnancy with respect to cost-effectiveness, potential to reduce health care costs and system burden, and their potential positive effect on maternal and neonatal outcomes [19,45]. Since the MediMama app is freely accessible and operates without a profit-generating model, the lack of secured funding poses a significant risk to its ongoing maintenance and sustainability, potentially jeopardizing its continued availability. Further research is needed to identify sustainable approaches to integrate these interventions within maternity care to ensure their continued maintenance, updating, and optimization. In addition, to reach the entire Dutch population of pregnant and breastfeeding women, establishing and maintaining long-term collaboration with health care professionals, such as midwives and gynecologists, is critical. While the target user group changes over time, health care professionals represent a stable and trusted gateway to this population. Further evaluation of effective strategies to continuously reach the different target populations is warranted. Finally, since the multiplicity of mobile apps in pregnancy can be overwhelming and choosing the best one is proven to be difficult for pregnant women, future efforts should explore unified, multilingual platforms that balance usability with comprehensive, evidence-based content [61].

### Conclusion

This study describes the development and dissemination process of the MediMama app, designed to provide easily accessible and evidence-based safety information on OTC medicines during pregnancy and breastfeeding. One year postlaunch, MediMama has been a well-received and frequently used eHealth resource in the Netherlands, effectively meeting the information needs of its target population. While initial feedback and usage patterns of MediMama are promising, the behavioral impact of the app remains untested, and its usability has yet to be formally assessed, warranting further evaluation. Further research is required to evaluate the effectiveness of the implementation strategy, as well as the app’s impact on maternal medication use behaviors and health outcomes. Mobile health is emerging as a pivotal tool in maternity care; increased emphasis is required on the continued development of these tools to improve the health of future generations.

## Abbreviations:

eHealth: electronic health
OTC: over-the-counter
TIS: Teratology Information Service
